# Macular Choroidal Small-Vessel Layer, Sattler’s Layer and Haller’s Layer Thicknesses: The Beijing Eye Study

**DOI:** 10.1038/s41598-018-22745-4

**Published:** 2018-03-13

**Authors:** Jing Zhao, Ya Xing Wang, Qi Zhang, Wen Bin Wei, Liang Xu, Jost B. Jonas

**Affiliations:** 1 Beijing Institute of Ophthalmology and Beijing Ophthalmology and Visual Science Key Lab, Beijing Tongren Eye Center, Beijing Tongren Hospital, Capital Medical University, Beijing, China; 2 Beijing Tongren Eye Center, Beijing Tongren Hospital, Capital Medical University, Beijing, China; 3 Department of Ophthalmology, Medical Faculty Mannheim, Ruprecht-Karls-University Heidelberg, Heidelberg, Germany

## Abstract

To study macular choroidal layer thickness, 3187 study participants from the population-based Beijing Eye Study underwent spectral-domain optical coherence tomography with enhanced depth imaging for thickness measurements of the macular small-vessel layer, including the choriocapillaris, medium-sized choroidal vessel layer (Sattler’s layer) and large choroidal vessel layer (Haller’s layer). In multivariate analysis, greater thickness of all three choroidal layers was associated (all *P* < 0.05) with higher prevalence of age-related macular degeneration (AMD) (except for geographic atrophy), while it was not significantly (all *P* > 0.05) associated with the prevalence of open-angle glaucoma or diabetic retinopathy. There was a tendency (0.07 > *P* > 0.02) toward thinner choroidal layers in chronic angle-closure glaucoma. The ratio of small-vessel layer thickness to total choroidal thickness increased (*P* < 0.001; multivariate analysis) with older age and longer axial length, while the ratios of Sattler’s layer and Haller’s layer thickness to total choroidal thickness decreased. A higher ratio of small-vessel layer thickness to total choroidal thickness was significantly associated with a lower prevalence of AMD (early type, intermediate type, late geographic type). Axial elongation-associated and aging-associated choroidal thinning affected Haller’s and Sattler’s layers more markedly than the small-vessel layer. Non-exudative and exudative AMD, except for geographic atrophy, was associated with slightly increased choroidal thickness.

## Introduction

Based on histological examinations performed by anatomists such as Sattler and Haller as early as 1876, the choroid has been stratified into three layers: the choriocapillaris, forming the inner layer and contributing its basement membrane to the structure of Bruch’s membrane; the middle choroidal layer, also called Sattler’s layer and including the middle-sized choroidal blood vessels; and the outer choroidal layer, also called Haller’s layer and containing the large choroidal blood vessels^1–3^. A fourth element in the choroidal compartment can be the suprachoroidal space if the choroid detaches from the sclera in conditions such as ocular hypotony. The choroid has been described by Nickla and Wallman as a multifunctional tissue, including vascular supply to the outer retina, temperature regulation, modulation of vascularization and growth of the sclera, and they focusing on some of the functions attributed to it^3^. Since Spaide and colleagues described in their landmark study the technique for visualization and semi-quantitative assessment of the choroid by applying the enhanced depth imaging mode of spectral-domain optical coherence tomography (OCT), numerous investigations have focused on measuring the thickness of the choroid as a whole and assessing the associations between total choroidal thickness and other ocular and systemic parameters^4–6^.

The spatial resolution of spectral-domain OCT also allows for visualization of the medium-sized and small-sized blood vessels in the choroid so that the choroid can be stratified into Haller’s layer, Sattler’s layer, and the remaining small-vessel layer, which also includes the choriocapillaris. Previous studies have demonstrated the involvement of the choroid in ocular diseases, such as central serous choroidopathy, polypoidal choroidal vasculopathy, Vogt-Koyanagi-Harada’s disease, choroidal neovascularization and the late stage of age-related macular degeneration (AMD), to mention only a few^7–10^. Since the involvement of the choroid in diseases can affect the choroidal layers differently, we conducted this study to measure the thickness of the three choroidal layers in normal eyes and in eyes with ocular diseases. To reduce the risk of selection bias, we chose population-based recruitment of the study participants.

## Materials and Methods

The Beijing Eye Study 2011 is a population-based prospective cohort study performed in northern China that has recently been described in detail^11–13^. According to the Declaration of Helsinki, the Medical Ethics Committee of the Beijing Tongren Hospital approved the study, and all of the participants provided informed written consent. The ethics committee confirmed that all off the methods were performed in accordance with the relevant guidelines and regulations. The study was performed in 5 communities in the urban district of Haidian in the north of Central Beijing and in 3 communities in the village area of Yufa of the Daxing District south of Beijing. The only eligibility criterion for inclusion into the study was an age of 50 + years old. In 2011, the 8 communities had a total population of 4403 individuals aged 50 years or older. Of the 4403 eligible subjects, 3468 individuals (1963 (56.6%) women) participated in eye examinations, corresponding to an overall response rate of 78.8%. The study was divided into rural (1633 (47.1%) subjects; 943 (57.7%) women) and urban parts (1835 (52.9%) subjects; 1020 (55.6%) women). The mean age was 64.6 ± 9.8 years old (median: 64 years; range 50–93 years).

All of the participants underwent standardized interviews with questions about their family status, level of education, income, quality of life, physical activity, psychic depression and known major systemic diseases. Blood pressure, body height and weight, and the circumferences of the waist and hip were measured. Fasting blood samples were collected for measurement of the concentrations of glucose, blood lipids and glycosylated hemoglobin. The ophthalmic examinations included measurement of automatic refractometry (Auto Refractometer AR-610; Nidek Co., Ltd., Tokyo, Japan), best-corrected visual acuity, tonometry (CT-60 computerized tonometer; Topcon Ltd., Tokyo, Japan), slit-lamp based biomicroscopy of the anterior and posterior segments of the eyes, optical low-coherence reflectometry (Lensstar 900 Optical Biometer, Haag-Streit, 3098 Koeniz, Switzerland) for biometry of the right eyes and for digital photography of the cornea and lens (Neitz Instruments Co, Tokyo, Japan) and of the optic disc and macula (Type CR6–45NM, Canon Inc., Lake Success, NY, USA). Using the Mini-Mental State Examination or Folstein test, we assessed cognitive function^14^.

**Figure 1:**
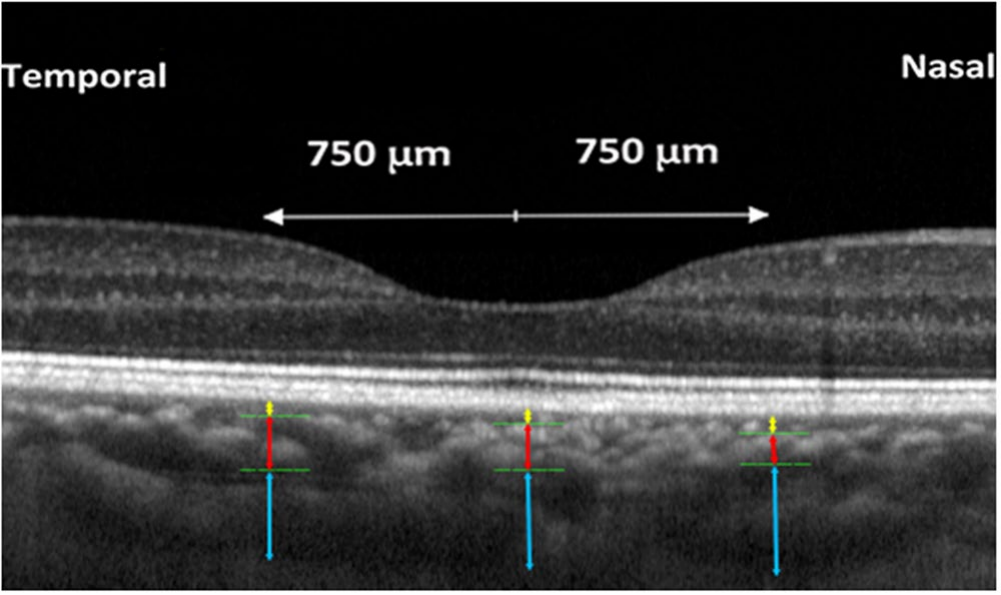
Optical coherence tomographic horizontal image of the macula showing the assessment of the thickness of the large vessel layer (Haller’s layer, outer layer), the medium-sized vessel layer (Sattler’s layer; middle layer), and the remaining inner layer containing small vessels, including the choriocapillaris, in the foveal region and at 750 µm nasal and temporal to the foveola.

Using the enhanced depth imaging modality of spectral-domain OCT (Spectralis^®^, Heidelberg Engineering Co., Heidelberg, Germany), we visualized and delineated the three choroidal layers, i.e., the small-vessel layer, Sattler’s layer and Haller’s layer, and we measured their thicknesses in the subfoveal region and at a distance of 750 μm nasal to the fovea and at a distance of 750 μm temporal to the fovea (Fig. 1). The choroid was defined as the distance from the choroidal-scleral interface to Bruch’s membrane. Haller’s layer was defined as the outer choroidal layer containing the large choroidal vessels. Sattler’s layer was defined as the layer of medium-sized choroidal vessels, which were visualized as medium-sized, hypo-intense spaces surrounded by a hyper-intensive stroma. A major criterion to differentiate between Sattler’s layer and Haller’s layer was the density of the inter-vascular tissue, including the choroidal melanocytes, which contributed to the scattering of the OCT signal within the choroidal stroma^15^. The remaining choroidal vessels on the inner side of Sattler’s layer consisted of small vessels, including the choriocapillaris immediately beneath the retinal pigment epithelium. The delineation line between the small-vessel layer and Sattler’s layer ran parallel to Bruch’s membrane. Applying the Heidelberg Eye Explorer software (v.5.3.3.0; Heidelberg Engineering Co, Heidelberg, Germany), we measured the thicknesses of all three choroidal layers on horizontally orientated OCT sections at the foveal region, at 750 μm nasal to the fovea and at 750 μm temporal to the fovea. Only the right eye of each study participant was measured. The OCT images were examined manually by two ophthalmologists (J.Z, Q.Z). This method of manually delineating the three choroidal layers is partially analogical to the automated choroidal blood vessel count and blood vessel area measurement as described by Chhablani and colleagues^16,17^.

We used a statistical software package (SPSS for Windows, version 22.0; SPSS, Inc., Chicago, IL, USA) for the statistical analysis. In a first step, we determined the mean values (presented as the mean ± standard deviation) of the thicknesses of different choroidal layers. In a second step, we performed univariate analyses of the associations between the thickness measurements and other ocular and systemic parameters. In a third step, we performed multivariate linear regression analysis, with the choroidal layer thickness as the dependent parameter and all the variables as independent parameters that were significantly associated with the choroidal thickness parameter in the univariate analysis. Odds ratios (ORs), standardized regression beta coefficients, the non-standardized B coefficients, and the 95% confidence intervals (CIs) were calculated. All *P*-values were two sided and were considered statistically significant if the values were less than 0.05. In a first approach, we included in the statistical analysis only a normal group consisting of eyes without glaucoma, AMD, diabetic retinopathy, retinal vein occlusions, polypoidal choroidal vasculopathy or central serous choroidopathy and with an axial length less than 26.5 mm. In a second approach, we assessed in the multivariate approach whether the prevalence of ocular disorders, the presence of which was an exclusion criterion for inclusion in the normal group, was correlated with the choroidal layer thickness determinations. To measure the inter-observer reproducibility and intra-observer reproducibility of the measurements, we randomly selected the images of 50 individuals. Two examiners (J.Z, Q.Z) repeated the measurements three times.

The datasets generated during and/or analyzed during the current study are available from the corresponding author on reasonable request.

## Results

Measurements of all three vessel layers were available for 3187 (91.9%) individuals (1786 (56.0%) women) of the 3468 participants of the Beijing Eye Study. The mean age was 64.0 ± 9.6 years old (median 63 years; range 50–93 years). The group of subjects without choroidal layer thickness measurements, compared with the group of subjects with measurements, was significantly (*P* < 0.001) older (71.1 ± 10.2 years versus 64.0 ± 9.6 years; *P* < 0.001) and more myopic (−2.7 ± 5.3 diopters versus −0.08 ± 1.8 diopters; *P* < 0.001). The main reason for not identifying and measuring the choroid layers was the poor quality of the OCT images, mainly due to signal attenuation (219 eyes; 6.3%), as found particularly in older eyes, pronounced thinning of the whole choroid in highly myopic eyes (38 eyes; 1.1%) so that the individual choroidal layers could no longer be distinguished, or other reasons (24 eyes; 0.7%).

The normal study group included 1992 individuals without glaucoma, AMD, diabetic retinopathy, retinal vein occlusions, polypoidal choroidal vasculopathy or central serous choroidopathy and with an axial length less than 26.5 mm. The mean age was 62.7 ± 9.3 years (median: 61 years, range: 50–93 years), and the mean axial length was 23.2 ± 1.0 mm (median: 23.1 mm, range: 18.96–26.47 mm). The mean thicknesses of the subfoveal small-vessel choroidal layer, medium-sized choroidal vessel layer, large choroidal vessel layer and total choroidal thickness were 30.5 ± 9.8 μm (median 28.0 μm; range 6.0 to 90.0 μm), 91.6 ± 39.0 μm (median 90.0 μm; range 12.0–281.0 μm), 155.2 ± 65.7 μm (median 151.0 μm; range 28.0–595.0 μm), and 277.2 ± 101.8 μm (median 274.0 μm; range 50.0–810.0 μm), respectively (Table 1). The thickness of the small-vessel layer combined with the medium-sized choroidal vessel layer was 122.1 ± 43.9 μm (range: 18.0–367.0 μm). The small-vessel layer thickness was significantly (*P* < 0.001) the greatest at the location of 750 μm temporal to the fovea, while the measurements obtained at the foveal region and at the location of 750 μm nasal to the fovea did not differ significantly (*P* = 0.054). The thickness of the medium-sized choroidal layer was significantly (*P* < 0.001) the greatest at the foveal region, followed by the location of 750 μm temporal to the fovea, where it was thicker (*P* < 0.001) than at 750 μm nasal to the fovea. The thickness of the large choroidal vessel layer was significantly (*P* = 0.001) thicker at the foveal region than at 750 μm temporal to the fovea, where it was thicker (*P* < 0.001) than at 750 μm nasal to the fovea (Table 1).

**Table 1:** Measurements of the various choroidal layers (mean ± standard deviation) in eyes without glaucoma, age-related macular degeneration, diabetic retinopathy, retinal vein occlusions, polypoidal choroidal vasculopathy or central serous choroidopathy and with an axial length less than 26.5 mm in the Beijing Eye Study 2011.

Thickness (μm)	Subfoveal	750 μm Nasal to the Fovea	750 μm Temporal to the Fovea
Small-Vessel Layer Including Choriocapillaris	30.5 ± 9.8	30.9 ± 10.3	32.6 ± 10.5
Medium-Sized Choroidal Vessel Layer (Sattler’s layer)	91.6 ± 39.0	85.1 ± 38.2	89.4 ± 36.7
Large-Sized Choroidal Vessel Layer (Haller’s layer)	155.2 ± 65.7	148.5 ± 65.3	152.9 ± 59.9
Total Choroid	277.2 ± 101.8	264.3 ± 102.6	274.9 ± 94.1
Ratio of Small-Vessel Layer (Including Choriocapillaris) Thickness to Total Choroidal Thickness	0.12 ± 0.04	0.13 ± 0.05	0.13 ± 0.04
Ratio of Medium-Sized Vessel Layer Thickness to Total Choroidal Thickness	0.33 ± 0.06	0.32 ± 0.06	0.32 ± 0.06
Ratio of Large-Sized Vessel Layer Thickness to Total Choroidal Thickness	0.55 ± 0.07	0.55 ± 0.07	0.55 ± 0.07

In univariate analysis, the mean thicknesses of the small-vessel layer, Sattler’s layer and Haller’s layer and of the total choroidal thickness were significantly associated with the systemic parameters of younger age (all *P* < 0.001), rural region of habitation (all *P* < 0.001), greater body weight (all *P* < 0.001) and higher body mass index (all *P* < 0.001), longer waist circumference (*P* = 0.03; *P* = 0.04* P* = 0.03; *P* = 0.01, resp.) and hip circumference (all *P* < 0.001), higher diastolic blood pressure (all *P* < 0.001), less aspirin intake (*P* = 0.02; *P* = 0.03; *P* < 0.001; and *P* < 0.001, resp.), higher blood concentration of glucose (*P* = 0.001; *P* = 0.001; *P* = 0.05; and *P* = 0.006, resp.), higher frequency of smoking (all *P* < 0.001) and greater number of cigarette pack-years (*P* = 0.05; *P* < 0.001; *P* < 0.001; and *P* < 0.001, resp.), lower prevalence of known cardiovascular disease (all *P* < 0.001), higher prevalence of hyperlipidemia (*P* = 0.05; *P* < 0.001; *P* < 0.001; and *P* < 0.001, resp.), and lower prevalence of previous cerebral infarction or hemorrhage (*P* = 0.001; *P* < 0.001; *P* < 0.001; and *P* < 0.001, resp.). The mean thicknesses of Sattler’s layer, Haller’s layer and total choroid but not the thickness of the small-vessel layer (*P* > 0.05) were significantly associated also with male gender (all *P* < 0.001), higher blood concentrations of low-density lipoproteins (*P* = 0.05; *P* = 0.02; and *P* = 0.02, resp.) and cholesterol (*P* = 0.04; *P* = 0.01; and *P* = 0.01, resp.), higher frequency of reported snoring (*P* < 0.001; *P* = 0.005; and *P* = 0.001, resp.), greater quantity of alcohol consumption (all *P* < 0.001) and higher cognitive function score (all *P* < 0.001).

The mean thicknesses of all three choroidal layers and of the total choroid were significantly correlated with the ocular parameters of hyperopic refractive error (all *P* < 0.001), better best-corrected visual acuity (measured in logMAR; all *P* < 0.001), shorter axial length (all *P* < 0.001), shorter corneal curvature radius (all *P* < 0.001), less anterior-chamber depth (all *P* < 0.001), higher prevalence of glaucoma (*P* = 0.01; *P* = 0.001; *P* = 0.004; and *P* = 0.001, respectively) and greater retinal nerve fiber layer thickness (all *P* < 0.001). Greater thicknesses of Sattler’s layer and Haller’s layer and of the total choroid were significantly associated with thinner lens (all *P* = 0.001) and higher intraocular pressure (all *P* < 0.001), while the mean thickness of the small-vessel layer was not significantly associated (all *P* > 0.05) with these parameters. The thicknesses of all three choroidal layers were not significantly (all *P* > 0.05) associated with the systemic parameters of systolic blood pressure, pulse, serum concentrations of high-density lipoproteins and triglycerides, presence of diabetes mellitus, or the ocular parameters of corneal diameter, central corneal thickness and diabetic retinopathy.

The multivariate analysis included the thicknesses of the various choroidal layers as dependent variables and all of the parameters as independent variables, which were significantly (*P* < 0.05) associated with them in the univariate analysis. In a step-by-step approach, due to collinearity, we first dropped refractive error from the list of independent parameters. All of the parameters were then excluded that were no longer significantly associated with the choroidal thickness variables, starting with the parameters with the highest *P*-values. In the final model, thicker subfoveal small-vessel layer thickness was significantly (regression coefficient r: 0.26) associated with younger age (*P* < 0.001), shorter axial length (*P* < 0.001) and larger corneal curvature radius (*P* = 0.01) (Table 2). If all of the eyes in the Beijing Eye Study with thickness measurements of the various choroidal layers were included and added to the model, greater subfoveal small-vessel thickness was significantly associated a with higher prevalence of AMD (any type) (*P* = 0.02) and with a higher prevalence of the intermediate type (*P* = 0.001), late type (*P* = 0.007) and late neovascular type of AMD (*P* = 0.04), while it was not significantly associated with the prevalence of open-angle glaucoma (*P* = 0.89), angle-closure glaucoma (*P* = 0.06), chronic retinal vein occlusion (*P* = 0.08), or diabetic retinopathy (*P* = 0.14) (Table 2).

**Table 2:** Multivariate analysis of associations between the subfoveal small-vessel layer thickness (including choriocapillaris) and systemic and ocular parameters in the Beijing Eye Study 2011, in eyes without glaucoma, age-related macular degeneration, diabetic retinopathy, retinal vein occlusions, polypoidal choroidal vasculopathy or central serous choroidopathy and with an axial length less than 26.5 mm (first three lines) or in the total study population (lines 4–15).

Parameter	*P*-Value	Standardized Regression Coefficient beta	Non- Standard. Regression Coefficient B	95% Confidence Interval	Variance Inflation Factor
Age (years)	<0.001	−0.17	−0.19	−0.23, −0.14	1.01
Axial Length (mm)	<0.001	−0.21	−2.19	−2.72, −1.62	1.49
Corneal Curvature Radius (mm)	0.01	0.07	2.69	0.64, 4.74	1.47
Disease					
Age-Related Macular Degeneration, Any Type	0.02	0.04	0.99	0.18, 1.81	1.04
Age-Related Macular Degeneration, Early Type	0.10	−0.03	−1.07	−2.33, 0.20	1.00
Age-Related Macular Degeneration, Intermediate Type	0.001	0.0.06	1.63	0.70, 2.57	1.03
Age-Related Macular Degeneration, Late Type	0.007	0.05	6.50	1.78, 11.2	1.01
Age-Related Macular Degeneration, Late Type, Geographic Atrophy	0.98	0.00	0.07	−5.53, 5.68	1.00
Age-Related Macular Degeneration, Late Type, Neovascular Type	0.04	0.04	0.55	0.01, 1.09	1.02
Open-Angle Glaucoma	0.89	−0.003	−0.16	−2.35, 2.03	1.02
Angle-Closure Glaucoma	0.06	−0.03	3.38	−6.85, −0.09	1.01
Chronic Retinal Vein Occlusion	0.08	−0.03	−2.79	−5.86, 0.28	1.01
Diabetes Mellitus	0.34	0.02	0.51	−0.54, 1.55	1.03
Diabetic Retinopathy	0.14	0.03	1.73	−0.57, 4.02	1.01
Cognitive Function Score	0.67	−0.01	0.02	−0.13, 0.09	1.08

Greater thickness of the subfoveal Sattler’s layer was significantly (r: 0.48) correlated in the multivariate model with younger age (*P* < 0.001), male gender (*P* < 0.001), shorter axial length (*P* < 0.001), and longer corneal curvature radius (*P* < 0.001) (Table 3). If all of the eyes in the Beijing Eye Study with thickness measurements of the various choroidal layers were included and added to the model, the thickness of the subfoveal Sattler’s layer was significantly associated with a higher prevalence of AMD (any type) (*P* < 0.001), the early type (*P* = 0.01), intermediate type (*P* < 0.001) and neovascular type of AMD (*P* = 0.002), lower prevalence of angle-closure glaucoma (*P* = 0.04) and higher prevalence of diabetes mellitus (*P* = 0.008), while it was not significantly associated with the prevalence of open-angle glaucoma (*P* = 0.30), chronic retinal vein occlusion (*P* = 0.34), or diabetic retinopathy (*P* = 0.24) (Table 3).

**Table 3:** Multivariate analysis of associations between the thickness of the subfoveal medium-sized choroidal vessel layer (Sattler’s layer) and systemic and ocular parameters in the Beijing Eye Study 2011, in eyes without glaucoma, age-related macular degeneration, diabetic retinopathy, retinal vein occlusions, polypoidal choroidal vasculopathy or central serous choroidopathy and with an axial length less than 26.5 mm (first three lines) or in the total study population (lines 4–15).

Parameter	*P*-Value	Standardized Regression Coefficient beta	Non- Standardized Regression Coefficient B	95% Confidence Interval	Variance Inflation Factor
Age (years)	<0.001	−0.36	−1.54	−1.71, −1.37	1.01
Gender	<0.001	−0.15	−11.6	−14.8, −8.32	1.09
Axial Length(mm)	<0.001	−0.34	−13.8	−15.9, −11.8	1.54
Corneal Curvature Radius (mm)	<0.001	0.11	16.2	8.8, 23.6	1.48
Disease					
Age-Related Macular Degeneration, Any Type	<0.001	0.09	8.36	5.48, 11.2	1.04
Age-Related Macular Degeneration, Early Type	0.01	0.03	3.77	−0.75, 8.28	1.00
Age-Related Macular Degeneration, Intermediate Type	<0.001	0.08	8.60	5.27, 11.9	1.03
Age-Related Macular Degeneration, Late Type	0.18	0.02	11.6	−5.27, 28.5	1.01
Age-Related Macular Degeneration, Late Type, Geographic Atrophy	0.13	−0.02	−15.4	−35.4, 4.58	1.00
Age-Related Macular Degeneration, Late Type, Neovascular Type	0.002	0.05	13.6	5.15, 22.0	1.01
Open-Angle Glaucoma	0.30	−0.02	−4.10	−11.9, 3.72	1.02
Angle-Closure Glaucoma	0.04	−0.03	−13.2	−25.5, −0.78	1.01
Chronic Retinal Vein Occlusion	0.34	−0.02	−5.35	−16.3, 5.59	1.01
Diabetes Mellitus	0.008	0.05	4.97	1.28, 8.67	1.03
Diabetic Retinopathy	0.24	0.02	4.91	−3.27, 13.1	1.01
Cognitive Function Score	0.03	0.04	0.43	0.04, 0.82	1.09

Combining the thickness of the small-vessel layer and the thickness of the medium-sized choroidal vessel layer showed that thicker combined thickness was associated with younger age (*P* < 0.001; beta: −0.36; B: −1.73; 95% CI: −1.92, −1.54), male gender (*P* < 0.001; beta: −0.14; B: −12.2; 95% CI: −15.8, −8.6), shorter axial length (*P* < 0.001; beta: −0.35; B: −16.1; 95% CI: −18.4, −13.8), and longer corneal curvature radius (*P* < 0.001; beta: 0.11; B: 18.8; 95% CI: 10.5, 27.1). After including all the eyes in the Beijing Eye Study, thicker combined small-vessel layer and medium-sized choroidal vessel layer was associated with a higher prevalence of AMD (any type) (*P* < 0.001; beta: 0.08; B: 8.45; 95% CI: 4.78, 12.1), intermediate AMD type (*P* < 0.001; beta: 0.09; B: 11.0; 95% CI: 6.78, 15.2), the neovascular type of AMD (*P* = 0.004; beta: 0.05; B: 16.0; 95% CI: 5.15, 26.8), and higher prevalence of diabetes mellitus (*P* = 0.01; beta: 0.05; B: 5.46; 95% CI: 1.30, 9.62), while it was not significantly associated with the prevalence of angle-closure glaucoma (*P* = 0.08), open-angle glaucoma (*P* = 0.54), the early type of AMD (*P* = 0.67), chronic retinal vein occlusion (*P* = 0.16), and diabetic retinopathy (*P* = 0.59).

Greater thickness of the subfoveal Haller’s layer was significantly (r: 0.50) associated in the multivariate analysis with younger age (*P* < 0.001), male gender (*P* < 0.001), shorter axial length (*P* < 0.001) and longer corneal curvature radius (*P* < 0.001) (Table 4). If all of the eyes in the Beijing Eye Study with thickness measurements of the various choroidal layers were included and added to the model, a thicker subfoveal Haller’s layer was significantly associated with a higher prevalence of AMD (any type) (*P* < 0.001) and with the early type (*P* = 0.02), intermediate type (*P* < 0.001) and late neovascular type of AMD (*P* < 0.001), as well as lower prevalence of chronic retinal vein occlusion (*P* = 0.04) and higher prevalence of diabetes mellitus (*P* = 0.01), while it was not significantly associated with the prevalence of late type of AMD (*P* = 0.05), open-angle glaucoma (*P* = 0.93), angle-closure glaucoma (*P* = 0.07), or diabetic retinopathy (*P* = 0.22) (Table 4). If cognitive function was added to the model, a thicker Haller’s layer was correlated with a higher cognitive function score (*P*

**Table 4:** Multivariate analysis of associations between the thickness of the subfoveal large choroidal vessel layer (Haller’s layer) and systemic and ocular parameters in the Beijing Eye Study 2011, in eyes without glaucoma, age-related macular degeneration, diabetic retinopathy, retinal vein occlusions, polypoidal choroidal vasculopathy or central serous choroidopathy and with an axial length less than 26.5 mm (first three lines) or in the total study population (lines 4–15).

Parameter	*P*- Value	Standardized Regression Coefficient beta	Non- Standardized Regression Coefficient B	95% Confidence Interval	Variance Inflation Factor
Age (years)	<0.001	−0.36	−1.54	−1.71, −1.37	1.01
Gender	<0.001	−0.15	−11.6	−14.8, −8.32	1.09
Axial Length (mm)	<0.001	−0.34	−13.8	−15.9, −11.8	1.54
Corneal Curvature Radius (mm)	<0.001	0.11	16.2	8.85, 23.6	1.48
Disease					
Age-Related Macular Degeneration, Any Type	<0.001	0.09	13.0	8.32, 17.8	1.04
Age-Related Macular Degeneration, Early Type	0.02	0.04	8.97	1.59, 16.3	1.00
Age-Related Macular Degeneration, Intermediate Type	<0.001	0.07	11.4	5.96, 16.9	1.03
Age-Related Macular Degeneration, Late Type	0.05	0.03	27.2	−0.39, 54.8	1.01
Age-Related Macular Degeneration, Late Type, Geographic Atrophy	0.47	−0.01	−12.0	−44.7, 20.7	1.00
Age-Related Macular Degeneration, Late Type, Neovascular Type	0.005	0.05	19.9	6.19, 33.8	1.01
Open-Angle Glaucoma	0.93	−0.001	−0.61	−13.4, 12.2	1.02
Angle-Closure Glaucoma	0.07	−0.03	−18.7	−38.9, 1.56	1.01
Chronic Retinal Vein Occlusion	0.04	−0.03	−19.1	−37.0, −1.28	1.01
Diabetes Mellitus	0.01	0.05	7.93	1.77, 14.1	1.03
Diabetic Retinopathy	0.22	0.02	8.44	−4.92, 21.8	1.01
Cognitive Function Score	0.002	0.05	1.01	0.37, 1.65	1.09

= 0.03).

Greater thickness of the total choroid layer was significantly (r: 0.52) associated with younger age (*P* < 0.001), male gender (*P* < 0.001), shorter axial length (*P* < 0.001) and longer corneal curvature radius (*P* < 0.001) (Table 5). If all of the eyes in the Beijing Eye Study with thickness measurements of the various choroidal layers were included and added to the model, greater subfoveal total choroidal thickness was significantly associated with a higher prevalence of AMD (any type) (*P* < 0.001), with the intermediate type (*P* < 0.001) and late neovascular type of AMD (*P* = 0.02) and with a lower prevalence of angle-closure glaucoma (*P* = 0.02), while it was not significantly associated with the prevalence of early type (*P* = 0.09) and late type of AMD (*P* = 0.35), open-angle glaucoma (*P* = 0.62), chronic retinal vein occlusion (*P* = 0.06) or diabetic retinopathy (*P* = 0.13) (Table 5). If cognitive function was added to the model, a thicker Haller’s layer was correlated with a higher cognitive function score (P = 0.002).

**Table 5:** Multivariate analysis of associations between the thickness of the whole subfoveal choroid and systemic and ocular parameters in the Beijing Eye Study 2011, in eyes without glaucoma, age-related macular degeneration, diabetic retinopathy, retinal vein occlusions, polypoidal choroidal vasculopathy or central serous choroidopathy and with an axial length less than 26.5 mm (first three lines) or in the total study population (lines 4–15).

Parameter	*P*- Value	Standardized Regression Coefficient beta	Non- Standardized Regression Coefficient B	95% Confidence Interval	Variance Inflation Factor
Age (years)	<0.001	−0.41	−4.45	−4.78, −4.11	1.01
Gender	<0.001	−0.16	−33.5	−40.2, −26.8	1.09
Axial Length (mm)	<0.001	−0.39	−39.2	−42.9, −35.4	1.50
Corneal Curvature Radius (mm)	<0.001	0.09	36.5	21.4, 51.6	1.44
Disease					
Age-Related Macular Degeneration, Any Type	<0.001	0.08	19.4	12.1, 26.6	1.04
Age-Related Macular Degeneration, Early Type	0.09	0.03	9.74	−1.69, 21.1	1.00
Age-Related Macular Degeneration, Intermediate Type	<0.001	0.07	19.6	11.2, 27.9	1.03
Age-Related Macular Degeneration, Late Type	0.35	0.02	20.2	−22.2, 62.4	1.01
Age-Related Macular Degeneration, Late Type, Geographic Atrophy	0.16	−0.02	−35.7	−85.7, 14.3	1.00
Age-Related Macular Degeneration, Late Type, Neovascular Type	0.02	0.04	25.3	4.22, 46.4	1.01
Open-Angle Glaucoma	0.62	−0.01	−5.00	−24.8, 14.8	1.02
Angle-Closure Glaucoma	0.02	−0.04	−37.2	−69.1, −5.25	1.01
Chronic Retinal Vein Occlusion	0.06	−0.03	−26.3	53.6, 1.05	1.01
Diabetes Mellitus	0.01	0.05	12.3	3.01, 21.6	1.03
Diabetic Retinopathy	0.13	0.02	16.0	−4.53, 36.6	1.01
Cognitive Function Score	<0.001	0.06	1.82	0.84, 2.80	1.09

The results of all three choroidal vascular layers and total choroidal thickness did not vary markedly if choroidal thickness measurements were used that were obtained subfoveally, nasally or temporally to the foveola.

**Figure 2:**
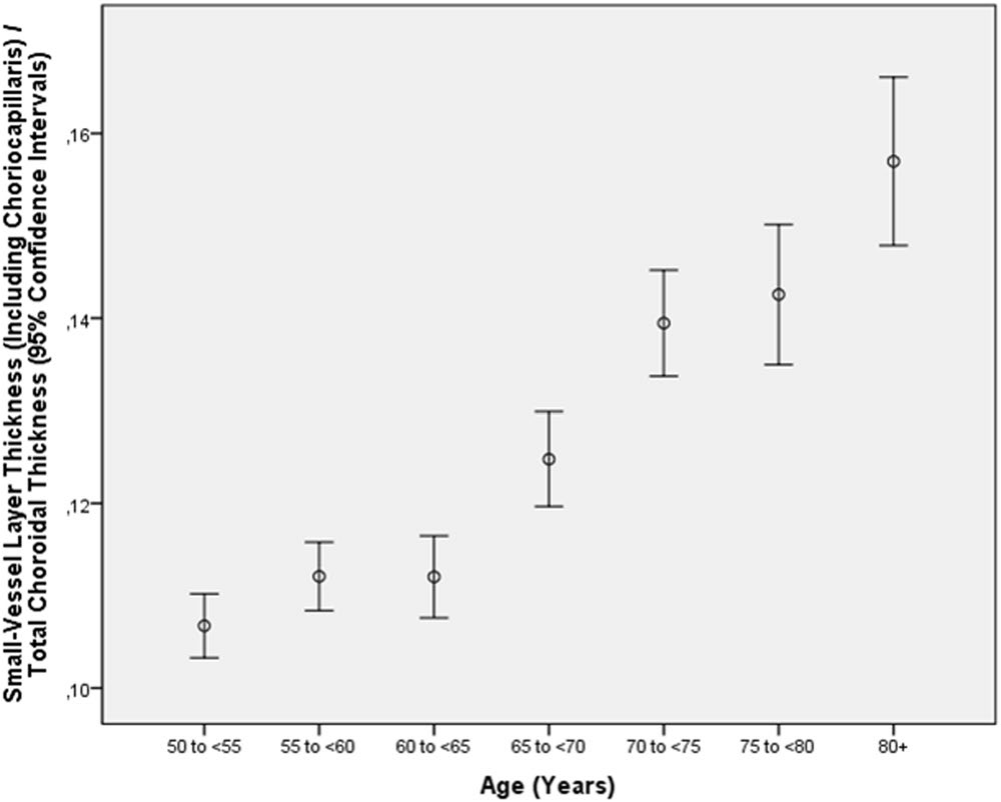
Graph showing the distribution of the ratio of subfoveal small-vessel layer thickness (including choriocapillaris) to total subfoveal choroidal thickness, stratified by age, in the Beijing Eye Study 2011 in eyes without glaucoma, age-related macular degeneration, diabetic retinopathy, retinal vein occlusions, polypoidal choroidal vasculopathy or central serous choroidopathy and with an axial length less than 26.5 mm.

**Figure 3:**
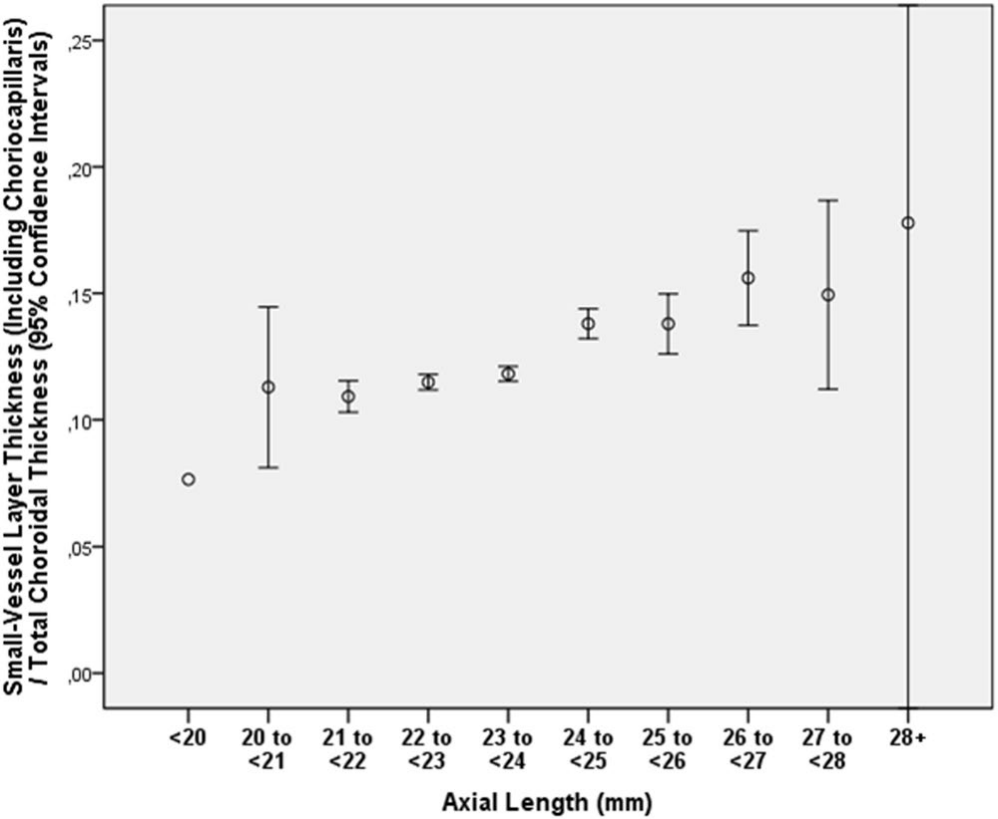
Graph showing the distribution of the ratio of subfoveal small-vessel layer thickness (including choriocapillaris) to total subfoveal choroidal thickness, stratified by axial length, in the Beijing Eye Study 2011 in eyes without glaucoma, age-related macular degeneration, diabetic retinopathy, retinal vein occlusions, polypoidal choroidal vasculopathy or central serous choroidopathy and with an axial length less than 26.5 mm.

**Figure 4:**
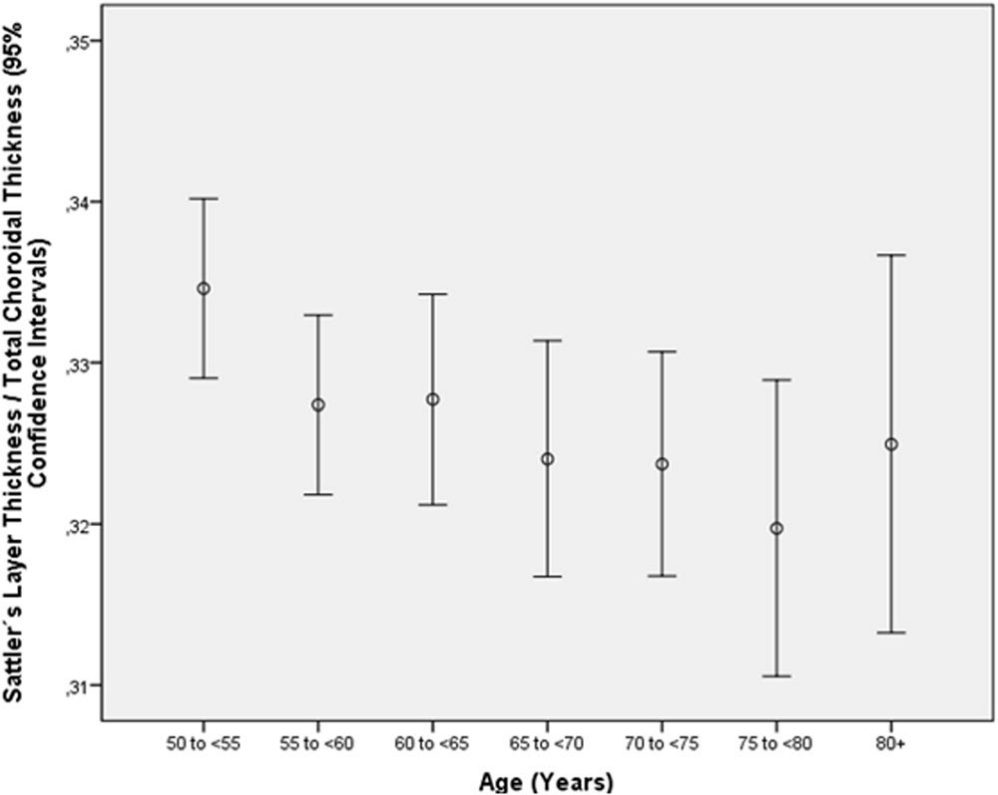
Graph showing the distribution of the ratio of thickness of the subfoveal medium-sized choroidal vessel layer (Sattler’s layer) to total subfoveal choroidal thickness, stratified by age, in the Beijing Eye Study 2011 in eyes without glaucoma, age-related macular degeneration, diabetic retinopathy, retinal vein occlusions, polypoidal choroidal vasculopathy or central serous choroidopathy and with an axial length less than 26.5 mm.

**Figure 5:**
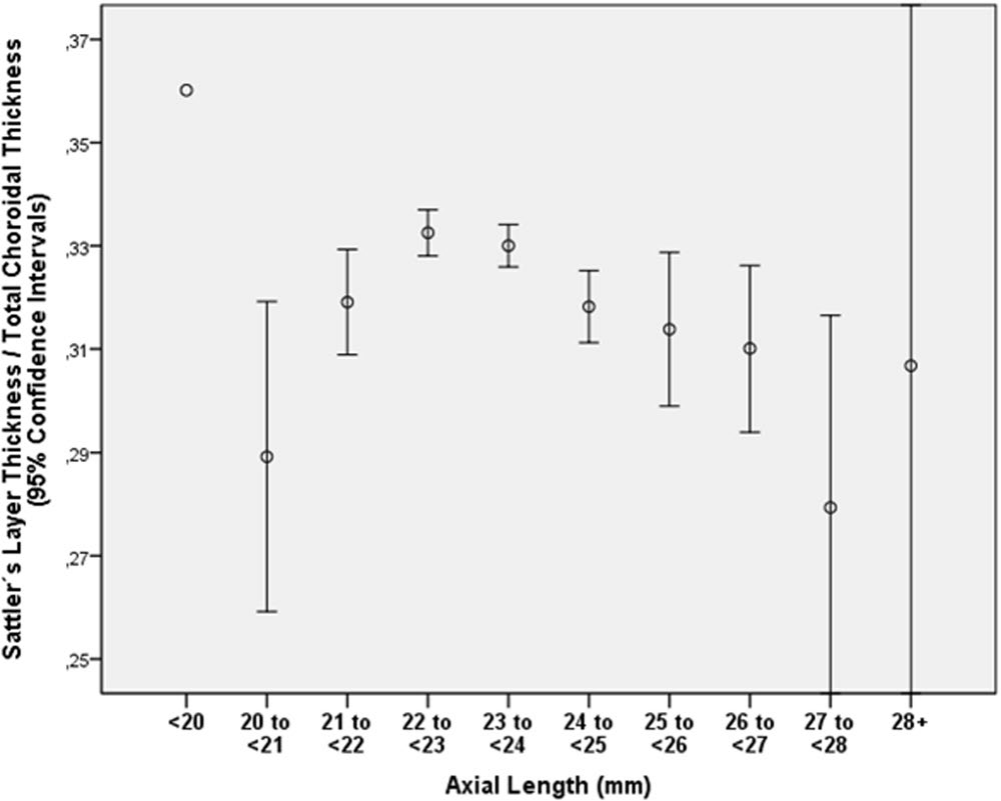
Graph showing the distribution of the ratio of thickness of the subfoveal medium-sized choroidal vessel layer (Sattler’s layer) to total subfoveal choroidal thickness, stratified by axial length, in the Beijing Eye Study 2011 in eyes without glaucoma, age-related macular degeneration, diabetic retinopathy, retinal vein occlusions, polypoidal choroidal vasculopathy or central serous choroidopathy and with an axial length less than 26.5 mm.

**Figure 6:**
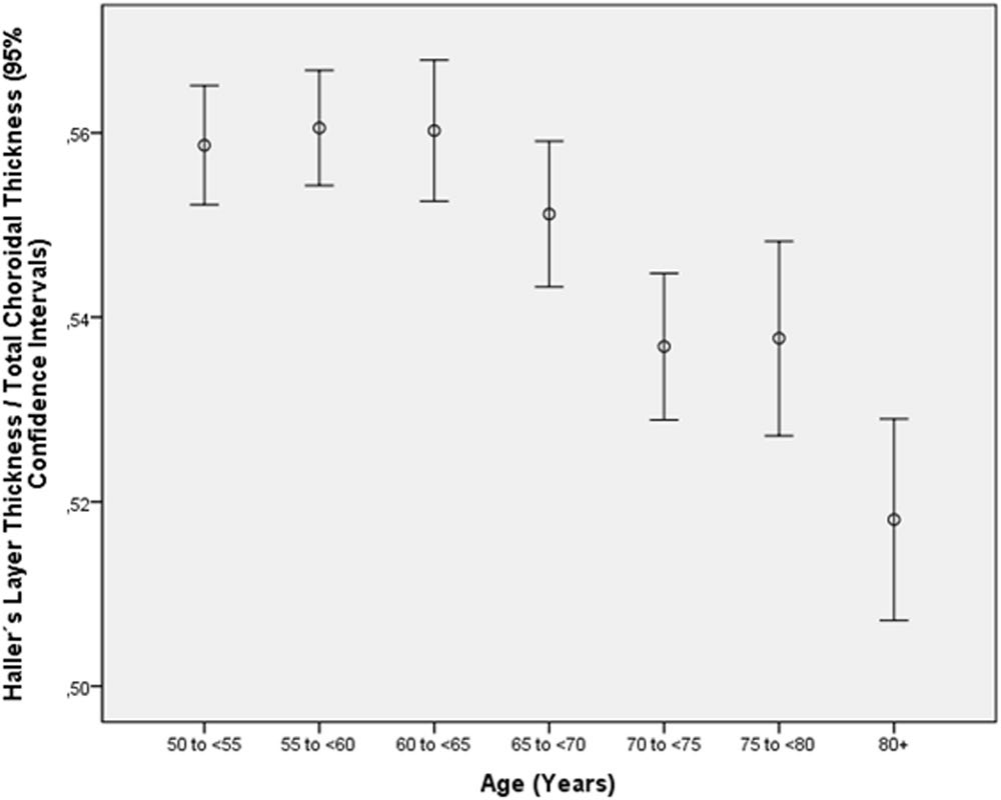
Graph showing the distribution of the ratio of thickness of the subfoveal large choroidal vessel layer (Haller’s layer) to total subfoveal choroidal thickness, stratified by age, in the Beijing Eye Study 2011 in eyes without glaucoma, age-related macular degeneration, diabetic retinopathy, retinal vein occlusions, polypoidal choroidal vasculopathy or central serous choroidopathy and with an axial length less than 26.5 mm.

**Figure 7:**
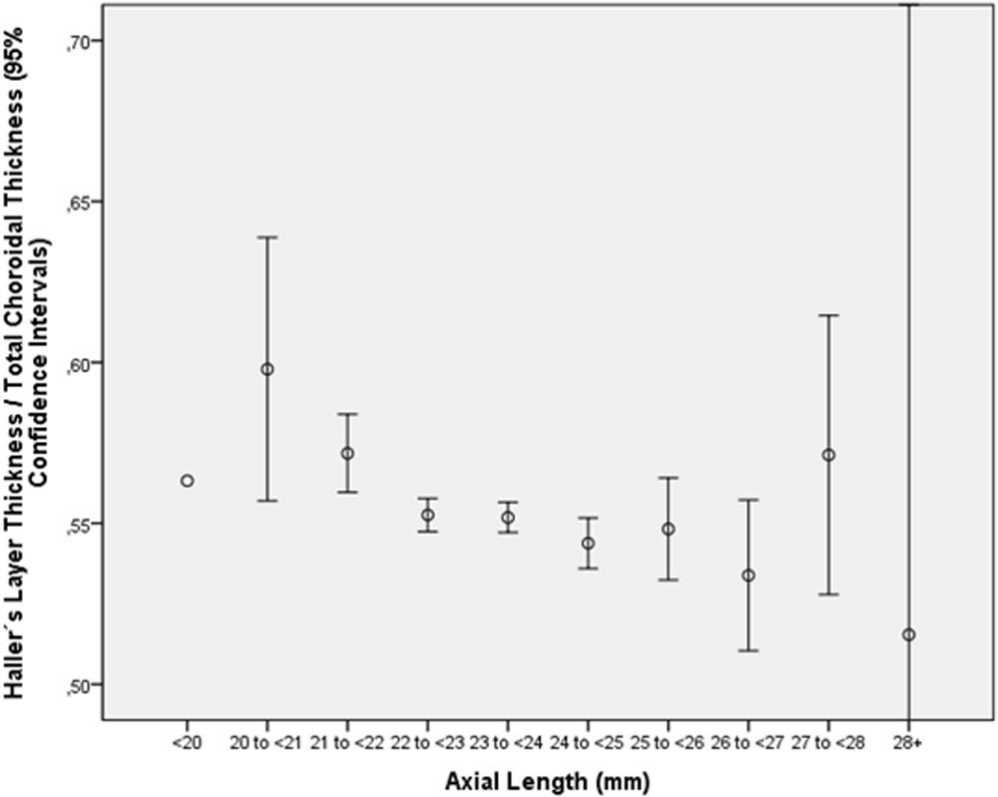
Graph showing the distribution of the ratio of thickness of the subfoveal large choroidal vessel layer (Haller’s layer) to total subfoveal choroidal thickness, stratified by axial length, in the Beijing Eye Study 2011 in eyes without glaucoma, age-related macular degeneration, diabetic retinopathy, retinal vein occlusions, polypoidal choroidal vasculopathy or central serous choroidopathy and with an axial length less than 26.5 mm.

The ratio of the small-vessel layer thickness to total choroidal thickness increased in multivariate analysis with older age (*P* < 0.001) and longer axial length (*P* < 0.001) (Table 6) (Figs 2 and 3). In contrast to the ratio of small-vessel layer thickness to total choroidal thickness, the ratio of Sattler’s layer thickness to total choroidal thickness decreased with older age (*P* = 0.007; beta: −0.06; B: −0.000; 95%CI: −0.001, 0.000) and with longer axial length (*P* = 0.003; beta: −0.07; B: −0.004; 95%CI: −0.006, −0.001) (Table 7) (Figs 4 and 5). Additionally, the ratio of Haller’s layer thickness to total choroidal thickness decreased with older age (*P* < 0.001; beta: −0.15; B: −0.001; 95%CI: −0.001, −0.001) and with longer axial length (*P* = 0.005; beta: −0.06; B: −0.004; 95%CI: −0.007, −0.001) (Table 8) (Figs 6 and 7). The ratio of small-vessel layer thickness to total choroidal thickness was significantly associated with a lower prevalence of AMD (early type, intermediate type, late geographic type) but not with the prevalence of any other disease examined (Table 6). The ratio of the thickness of Sattler’s layer or Haller’s layer to total choroidal thickness was not significantly correlated with the prevalence of any disorder examined (Tables 7 and 8).

**Table 6:** Multivariate analysis of associations between the ratio of subfoveal small-vessel layer thickness (including choriocapillaris) to total subfoveal choroidal thickness and systemic and ocular parameters in the Beijing Eye Study 2011, in eyes without glaucoma, age-related macular degeneration, diabetic retinopathy, retinal vein occlusions, polypoidal choroidal vasculopathy or central serous choroidopathy and with an axial length less than 26.5 mm (first three lines) or in the total study population (lines 4–15).

Parameter	*P*-Value	Standardized Regression Coefficient beta	Non- Standard. Regression Coefficient B	95% Confidence Interval	Variance Inflation Factor
Age (years)	<0.001	0.34	0.002	0.001, 0.002	1.01
Gender	<0.001	0.10	0.009	0.006, 0.012	1.09
Axial Length (mm)	<0.001	0.25	0.01	0.009, 0.012	1.50
Corneal Curvature Radius (mm)	0.003	−0.06	−0.010	−0.017, −0.003	1.44
Disease					
Age-Related Macular Degeneration, Any Type	0.001	−0.06	−0.006	−0.009, −0.003	1.04
Age-Related Macular Degeneration, Early Type	0.004	−0.05	−0.008	−0.013, −0.002	1.00
Age-Related Macular Degeneration, Intermediate Type	0.04	−0.04	−0.004	−0.008, 0.000	1.03
Age-Related Macular Degeneration, Late Type	0.39	0.01	0.008	−0.011, 0.028	1.01
Age-Related Macular Degeneration, Late Type, Geographic Atrophy	0.04	0.04	0.024	0.002, 0.047	1.00
Age-Related Macular Degeneration, Late Type, Neovascular Type	0.56	−0.010	−0.003	−0.012, 0.007	1.01
Open-Angle Glaucoma	0.32	0.02	0.004	−0.004, 0.013	1.02
Angle-Closure Glaucoma	0.40	0.01	0.006	−0.008, 0.020	1.01
Chronic Retinal Vein Occlusion	0.56	0.01	0.004	−0.009, 0.016	1.01
Diabetes Mellitus	0.33	−0.02	−0.002	−0.006, 0.002	1.03
Diabetic Retinopathy	0.86	0.003	0.001	−0.008, 0.010	1.01

**Table 7:** Multivariate analysis of associations between the ratio of the thickness of the subfoveal medium-sized choroidal vessel layer (Sattler’s layer) to total subfoveal choroidal thickness and systemic and ocular parameters in the Beijing Eye Study 2011, in eyes without glaucoma, age-related macular degeneration, diabetic retinopathy, retinal vein occlusions, polypoidal choroidal vasculopathy or central serous choroidopathy and with an axial length less than 26.5 mm (first three lines) or in the total study population (lines 4–15).

Parameter	*P*-Value	Standardized Regression Coefficient beta	Non- Standard. Regression Coefficient B	95% Confidence Interval	Variance Inflation Factor
Age (years)	0.007	−0.05	0.000	−0.001, 0.000	1.01
Axial Length (mm)	<0.001	−0.08	−0.004	−0.006, −0.002	1.01
Disease					
Age-Related Macular Degeneration, Any Type	0.19	0.20	0.003	−0.002, 0.008	1.04
Age-Related Macular Degeneration, Early Type	0.83	−0.004	−0.001	−0.008, 0.007	1.00
Age-Related Macular Degeneration, Intermediate Type	0.08	0.03	0.005	−0.001, 0.011	1.03
Age-Related Macular Degeneration, Late Type	0.50	−0.01	−0.010	−0.038, 0.018	1.00
Age-Related Macular Degeneration, Late Type, Geographic Atrophy	0.02	−0.04	−0.04	−0.073, −0.006	1.00
Age-Related Macular Degeneration, Late Type, Neovascular Type	0.30	0.02	0.007	−0.007, 0.022	1.00
Open-Angle Glaucoma	0.23	−0.02	−0.008	−0.021, 0.005	1.02
Angle-Closure Glaucoma	0.30	−0.02	−0.011	−0.032, 0.010	1.01
Chronic Retinal Vein Occlusion	0.19	0.02	0.012	−0.006, 0.030	1.01
Diabetes Mellitus	0.31	0.02	0.003	−0.003, 0.009	1.03
Diabetic Retinopathy	0.99	0.00	0.000	−0.014, 0.014	1.01

**Table 8:** Multivariate analysis of associations between the ratio of the thickness of the subfoveal large choroidal vessel layer (Haller’s layer) to total subfoveal choroidal thickness and systemic and ocular parameters in the Beijing Eye Study 2011, in eyes without glaucoma, age-related macular degeneration, diabetic retinopathy, retinal vein occlusions, polypoidal choroidal vasculopathy or central serous choroidopathy and with an axial length less than 26.5 mm (first three lines) or in the total study population (lines 4–15).

Parameter	*P*-Value	Standardized Regression Coefficient beta	Non- Standard. Regression Coefficient B	95% Confidence Interval	Variance Inflation Factor
Age (years)	<0.001	−0.17	−0.001	−0.001, −0.001	1.01
Axial Length (mm)	0.002	−0.05	−0.004	−0.006, −0.001	1.01
Disease					
Age-Related Macular Degeneration, Any Type	0.31	0.02	0.003	−0.003, 0.008	1.04
Age-Related Macular Degeneration, Early Type	0.07	0.03	0.008	−0.001, 0.017	1.00
Age-Related Macular Degeneration, Intermediate Type	0.85	−0.004	−0.001	−0.007, 0.006	1.03
Age-Related Macular Degeneration, Late Type	0.77	0.005	0.005	−0.27, 0.037	1.00
Age-Related Macular Degeneration, Late Type, Geographic Atrophy	0.45	0.001	0.015	−0.023, 0.052	1.00
Age-Related Macular Degeneration, Late Type, Neovascular Type	0.76	−0.006	−0.002	−0.018, 0.013	1.00
Open-Angle Glaucoma	0.59	0.010	0.004	−0.011, 0.019	1.02
Angle-Closure Glaucoma	0.68	0.008	0.005	−0.018, 0.028	1.01
Chronic Retinal Vein Occlusion	0.14	−0.027	−0.015	−0.036, 0.005	1.01
Diabetes Mellitus	0.71	−0.008	−0.001	−0.008, 0.006	1.03
Diabetic Retinopathy	0.91	−0.002	−0.001	−0.016, 0.015	1.01

The analysis of intra-observer variability revealed an intra-class correlation coefficient (ICC) greater than 0.945 (*P* < 0.05), and the inter-observer ICC reproducibility value was greater than 0.977.

## Discussion

Our population-based study revealed that greater thickness of all three choroidal layers was associated with younger age, shorter axial length and longer corneal curvature radius. The ratio of the small-vessel layer thickness to total choroidal thickness increased with older age and longer axial length, while correspondingly, the ratios of Sattler’s layer thickness and Haller’s layer thickness to total choroidal thickness decreased with older age and with longer axial length. Non-exudative and exudative AMD, except for geographic atrophy, were associated with increased choroidal thickness, which was more marked for Haller’s layer than for the small-vessel layer. Correspondingly, a lower ratio of small-vessel layer thickness to total choroidal thickness was associated with a higher prevalence of AMD. Thickness of any choroidal layer was not significantly correlated with the prevalence of open-angle glaucoma or diabetic retinopathy. There was a tendency (*P* between 0.07 and 0.02) toward less thickness of all three choroidal layers in angle-closure glaucoma.

The results obtained in our study agree with the findings reported in previous investigations. Using the Cirrus high-definition OCT device, Branchini and colleagues examined 42 healthy individuals with a mean age of 51.6 years old and measured the large-choroidal vessel layer thickness and total choroidal thickness^18^. With a mean subfoveal choroidal thickness of 257 ± 76 μm, the mean thickness of the subfoveal large choroidal vessel layer was 204 ± 66 μm, and the thickness of the remaining medium choroidal vessel layer-choriocapillaris layer was 53 ± 21 μm. The ratio of large-choroidal vessel layer thickness to total choroidal thickness was 0.7 ± 0.1. After adjustment for the difference in age, with the participants of our study being older than those of Branchini’s investigation, the thickness of the large-vessel layer in Branchini’s study of 204 ± 66 μm was comparable to the value obtained in our study of 155.2 ± 65 μm. Additionally, the ratio of the large-vessel layer to total choroidal thickness (in Branchini’s study 0.7 versus 0.6 in our study) was comparable if one considers that this ratio decreased with older age. The results of our study also agreed with the findings of another previous investigation in which the total choroid was significantly thinner nasally than in the subfoveal region or in the region temporal to the foveola^19^. Interestingly, in our study, the small-vessel layer, including the choriocapillaris, was thinner in the subfoveal region than nasally or temporally to the foveola (Table 1).

The association between thinner total choroidal thickness in eyes with longer axial length and in older individuals has been described in numerous previous investigations^5,6,20^. Our study extends the previous findings referring total choroidal thickness to observations of the thickness of the three different layers of the choroid. Interestingly, the age-related thinning and axial elongation-associated thinning of the posterior choroid were less marked for the small-vessel layer than for Sattler’s layer or Haller’s layer. Correspondingly, the ratio of the small-vessel layer thickness to total choroidal thickness increased with older age and longer axial length (Figs 2–4). Similar findings were obtained in a study by Ruiz-Medrano and coworkers, who performed a retrospective data analysis of a prospective, cross-sectional investigation of 169 healthy eyes examined by swept-source optical coherence tomography and the refractive error, which ranged within ±3 diopters^21^. As in our study, the authors observed age-related choroidal thinning mostly at the expense of Haller’s layer, while also as in our study, there were no gender-related differences. The clinical meaning of the observation that the age-related thinning and axial elongation-associated thinning of the posterior choroid occurred more markedly in Sattler’s layer and Haller’s layer than in the small-vessel layer has yet to be assessed. The finding might indicate that the general thinning of the choroid with longer axial length, including in highly myopic eyes, affected more markedly Sattler’s layer and Haller’s layer than the small-vessel layer, which was relatively spared from age-related and axial elongation-related thickness changes (although it also showed axial elongation-associated and age-related thinning on absolute terms). Since the small-vessel layer, including the choriocapillaris, is the main choroidal layer supporting the retinal pigment epithelium and the photoreceptors, the finding might explain the observation made in a previous population-based study that best-corrected visual acuity was independent of axial length if eyes with myopic maculopathy were excluded^22^. The relative increase in the small-vessel layer thickness with axial elongation is paralleled by findings that the macular retinal pigment epithelium density, the length and thickness of the macular Bruch’s membrane and the macular retinal thickness were independent of axial length, and all of these parameters contribute to the observation of best-corrected visual acuity independent of myopic axial elongation in eyes without myopic maculopathy^23^. In agreement with this finding, Gupta and colleagues reported that highly myopic eyes had low total subfoveal choroidal thickness, but total choroidal thickness was not an independent predictor of visual acuity^24^.

The association between slightly greater thickness of the choroidal layers and higher prevalence of AMD (except for the late geographic type of AMD) agreed with findings obtained in previous investigations, in which eyes with AMD either showed greater total subfoveal choroidal thickness than a control group or did not differ in choroidal thickness from the control group^25^. In a retrospective, cross-sectional analysis of the Age-Related Eye Disease Study, choroidal thickness, after adjusting for age and refractive error, did not vary significantly among 154 eyes without AMD, 109 eyes with intermediate AMD and 62 eyes with advanced AMD^25^. Fitting with the observation made in our study that geographic atrophy was not associated with increased thickness of the choroid, Lindner and associates found, in a clinical study of 72 eyes with geographic atrophy and 37 control eyes, that the choroid was significantly thinner in the eyes with geographic atrophy^26^. The difference between the two studies might have been due to the relatively small number of eyes with geographic atrophy in our study. Corresponding to slightly increased choroidal thickness in the untreated eyes with AMD in our study, treatment studies reported a significant reduction in subfoveal choroidal thickness after intravitreal application of anti-vascular endothelial growth factor in eyes with typical neovascular AMD, compared to eyes with polypoidal choroidal vasculopathy^27^. In other studies differentiating among the three choroidal layers, the study sample sizes might have been too small for a detailed statistical analysis^28^.

Because previous investigation our study did not show a significant association between thickness of the total choroid and the prevalence of open-angle glaucoma, we extended these findings to the thicknesses of the three different layers of the choroid^29^. Interestingly, there was a tendency toward a thinner choroid as a whole (*P* = 0.02) and a thinner Sattler’s layer (*P* = 0.04) in eyes with chronic angle-closure glaucoma (Tables 2–5). Such a finding would agree with a previous histomorphometric study in which the choroid was significantly thinner in eyes with chronic angle-closure glaucoma than in a control group^30^. It would also agree with a recent investigation in which eyes with increases in intraocular pressure in a dark room adaptation test showed thinning of the choroid^31^. This finding is also in partial contrast to the results of studies on eyes with acute angle-closure glaucoma in which an abnormally thick choroid was reported^9,10^.

The finding obtained in our study that the thickness of all three choroidal layers was not significantly correlated with the prevalence of diabetic retinopathy was in partial agreement with observations made in previous investigations. In an investigation by Querques and colleagues of 63 consecutive diabetic patients and 21 age- and sex-matched healthy subjects, subfoveal total choroidal thickness did not differ significantly among diabetic individuals without retinopathy, with non-proliferative diabetic retinopathy or with non-proliferative diabetic retinopathy with clinically significant cystoid macular edema^32^. Total choroidal thickness was thinner in each diabetic group than in the control group, however. Kim and associates observed that total choroidal thickness increased with a higher degree of diabetic retinopathy and with the presence of diabetic macular edema, while it decreased if panretinal photocoagulation had been performed^33^. Esmaeelpour and associates examined 33 patients with diabetes mellitus type 1 and 20 healthy axial eye length- and age-matched subjects and found that total subfoveal choroidal thickness was significantly thinner in all of the diabetic groups (with or without diabetic retinopathy) than in the healthy eyes^34^. Regatieri and colleagues examined 49 patients with diabetes and 24 age-matched normal control subjects and found that the mean total subfoveal choroidal thickness was thinner in patients with diabetic macular edema or treated proliferative diabetic retinopathy than in normal subjects or subjects with non-proliferative diabetic retinopathy^35^. In their study, however, choroidal thickness was not corrected for its dependence on axial length. Vujosevic and coworkers examined 102 diabetic patients and 48 normal subjects and reported that the total subfoveal choroidal thickness decreased with increasing levels of diabetic retinopathy^36^. Control individuals and diabetic patients without diabetic retinopathy did not differ significantly in total subfoveal choroidal thickness. In light of the divergent results of previous investigations, one must consider that the study participants were recruited on a hospital basis and that the study samples of the previous studies were relatively small–too small for a detailed multivariate analysis. It is, however, mandatory to adjust choroidal thickness measurements for their dependence on axial length and age before additional associations are tested.

Interestingly, greater thickness of Sattler’s layer and Haller’s layer was associated with a higher cognitive function score in the multivariate analysis, extending the observation made in a previous assessment that total choroidal thickness was correlated with a higher cognitive function score after adjusting for age, axial length, gender, anterior corneal curvature radius, anterior-chamber depth, lens thickness, best-corrected visual acuity, and depression score^37^. These findings are paralleled by observations made by Gharbiya and colleagues, who reported thinner subfoveal total choroid thickness in 21 patients with mild to moderate Alzheimer’s disease than in 21 age-matched control individuals^38^.

Potential limitations of our study should be mentioned. First, differentiating between polypoidal choroidal vasculopathy and exudative AMD is mandatory if choroidal thickness is assessed since polypoidal choroidal vasculopathy is characterized by pachychoroid. Although we used fundus photographs and OCT images in our study for the diagnosis of AMD and its differentiation from polypoidal choroidal vasculopathy, it cannot be excluded that some eyes with polypoidal choroidal vasculopathy were misdiagnosed with exudative AMD. Since, however, the early and intermediate (non-exudative) stages of AMD were also associated with slightly greater thickness of the choroidal layers, the finding of a slightly thickened choroid in eyes with AMD might not have been confounded by a misdiagnosis of polypoidal choroidal vasculopathy. Second, we did not consider diurnal changes in choroidal thickness^39–41^. Third, choroidal thickness was measured only in the right eye of each study participant, so inter-eye differences and their associations with inter-eye differences in other parameters could not be assessed. Fourth, since our study included only Chinese participants, it might be discussed whether the results can directly transferred to individuals with other ethnic backgrounds.

In conclusion, the general process of choroidal thinning with longer axial length and older age more markedly affected Haller’s and Sattler’s layers than the small-vessel layer. Non-exudative and exudative AMD, except for geographic atrophy, was associated with slightly increased choroidal thickness and was more marked for Haller’s layer than for the small-vessel layer. Angle-closure glaucoma showed a tendency toward thinning of all of the choroidal layers, while open-angle glaucoma and diabetic retinopathy were not significantly correlated with the thickness of any choroidal layer.
